# Vasoactive intestinal peptide–VIPR2 signaling regulates tumor cell migration

**DOI:** 10.3389/fonc.2022.852358

**Published:** 2022-09-27

**Authors:** Satoshi Asano, Misa Yamasaka, Kairi Ozasa, Kotaro Sakamoto, Atsuko Hayata-Takano, Takanobu Nakazawa, Hitoshi Hashimoto, James A. Waschek, Yukio Ago

**Affiliations:** ^1^ Department of Cellular and Molecular Pharmacology, Graduate School of Biomedical and Health Sciences, Hiroshima University, Hiroshima, Japan; ^2^ School of Dentistry, Hiroshima University, Hiroshima, Japan; ^3^ Research and Development Department, Ichimaru Pharcos Company Limited, Gifu, Japan; ^4^ Laboratory of Molecular Neuropharmacology, Graduate School of Pharmaceutical Sciences, Osaka University, Osaka, Japan; ^5^ Molecular Research Center for Children’s Mental Development, United Graduate School of Child Development, Osaka University, Kanazawa University, Hamamatsu University School of Medicine, Chiba University, and University of Fukui, Osaka, Japan; ^6^ Laboratory of Molecular Biology, Department of Bioscience, Graduate School of Life Sciences, Tokyo University of Agriculture, Tokyo, Japan; ^7^ Division of Bioscience, Institute for Datability Science, Osaka University, Osaka, Japan; ^8^ Transdimensional Life Imaging Division, Institute for Open and Transdisciplinary Research Initiatives, Osaka University, Osaka, Japan; ^9^ Department of Molecular Pharmaceutical Science, Graduate School of Medicine, Osaka University, Osaka, Japan; ^10^ Department of Psychiatry and Biobehavioral Sciences, David Geffen School of Medicine, Semel Institute for Neuroscience and Human Behavior, University of California Los Angeles, Los Angeles, CA, United States

**Keywords:** breast cancer, cell migration, cytoskeleton, GPCR, phosphatidylinositol signaling, VIPR2

## Abstract

Phosphoinositide metabolism is critically involved in human cancer cell migration and metastatic growth. The formation of lamellipodia at the leading edge of migrating cells is regulated by metabolism of the inositol phospholipid PI(4,5)P_2_ into PI(3,4,5)P_3_. The synthesized PI(3,4,5)P_3_ promotes the translocation of WASP family verprolin homologous protein 2 (WAVE2) to the plasma membrane and regulates guanine nucleotide exchange factor Rac-mediated actin filament remodeling. Here, we investigated if VIPR2, a receptor for vasoactive intestinal peptide (VIP), has a potential role in regulating cell migration *via* this pathway. We found that silencing of *VIPR2* in MDA-MB-231 and MCF-7 human breast cancer cells inhibited VIP-induced cell migration. In contrast, stable expression of exogenous VIPR2 promoted VIP-induced tumor cell migration, an effect that was inhibited by the addition of a PI3-kinase (PI3K)γ inhibitor or a VIPR2-selective antagonist. VIPR2 stably-expressing cells exhibited increased PI3K activity. Membrane localization of PI(3,4,5)P_3_ was significantly attenuated by *VIPR2*-silencing. *VIPR2*-silencing in MDA-MB-231 cells suppressed lamellipodium extension; in VIPR2-overexpressing cells, VIPR2 accumulated in the cell membrane on lamellipodia and co-localized with WAVE2. Conversely, *VIPR2*-silencing reduced WAVE2 level on the cell membrane and inhibited the interaction between WAVE2, actin-related protein 3, and actin. These findings suggest that VIP–VIPR2 signaling controls cancer migration by regulating WAVE2-mediated actin nucleation and elongation for lamellipodium formation through the synthesis of PI(3,4,5)P_3_.

## Introduction

Cell migration is an evolutionarily conserved mechanism that plays a role in normal and pathogenic processes, including embryogenesis, immunity, angiogenesis, wound healing, and cancer metastases. Orchestrated cell motility is regulated by polarized intracellular signaling, which leads to the formation of protrusive structures, such as lamellipodia, at the leading edge of cells (1). Specific extracellular stimuli, including growth factors, cytokines, and chemokines, induce the formation of lamellipodia. In the case of signaling through chemokines and their G-protein-coupled receptors (GPCRs), GTP-bound Gαi directly activates SRK-like kinases, which upregulate phosphatidylinositol 3 kinase (PI3K) signaling (2–4). A well-established effector of the Gβγ subunits is PI3Kγ (5). Activated PI3Ks convert phosphatidylinositol 4,5-bisphosphate [PI(4,5)P_2_] into phosphatidylinositol 3,4,5-trisphosphate [PI(3,4,5)P_3_]. Therefore, both the α and βγ subunits are involved in the production of PI(3,4,5)P_3_, stimulating its rapid formation in the leading edge of migrating cells (4). PI(3,4,5)P_3_ promotes the translocation of WASP family verprolin homologous protein 2 (WAVE2) to the plasma membrane and regulates GEF-Rac-mediated actin filament remodeling (6). *In vitro*, PI(3,4,5)P_3_ directly binds WAVE2 with nanomolar affinity within the basic domain, and this mediates membrane phosphoinositide signaling (7). WAVE2 drives lamellipodium formation by enhancing actin nucleation *via* the actin-related protein 2 and 3 (ARP2/3) complex (8). Gain-of-function mutations in PI3K (i.e., mutations in the p110 catalytic subunit of PI3K) enhance the PI(3,4,5)P_3_ signaling pathway and are frequently found in breast cancers and other cancers (9, 10). These activated PI(3,4,5)P_3_ pathways induce cancer cell growth and motility, resulting in enhanced cancer cell migration and invasion (11, 12). Higher levels and co-expression of WAVE2 and ARP2 have also been found in human metastatic lung adenocarcinoma and breast carcinoma and are closely associated with poor patient outcome (13, 14).

Pituitary adenylate cyclase-activating polypeptide (PACAP) and the closely related neuropeptide vasoactive intestinal peptide (VIP), exhibit widespread expression in the central and peripheral nervous systems (15). Their receptors PAC1, VIPR1 and VIPR2 (also known as VPAC1 and VPAC2) are widely expressed in the brain but are also present in a multitude of peripheral target organs, including those of cardiovascular, renal, digestive, immune, endocrine, and reproductive systems (16). PACAP is an autocrine growth factor for some lung cancer cells (17, 18). The activated PAC1 receptor causes PI turnover, elevates cAMP, and increases the proliferation of lung cancer cells. Additionally, VIP stimulates growth of several tumors including breast, lung, pancreas, prostate, in addition to various central nervous system tumors (19–22). VIP-immunoreactivity occurs in a number of tumors, and VIPR1 is overexpressed, resulting in high densities, in numerous cancers including bladder, breast, colon, lung, pancreatic, and prostate cancers (22, 23), although there are some inconsistencies with other studies (24, 25). In contrast, VIPR2 has been less well-studied, but is present in thyroid, gastric and lung carcinomas, pancreatic adenocarcinomas, sarcomas and neuroendocrine tumors (23, 26, 27). Notably, increased *VIPR2* mRNA expression and/or *VIPR2* gene copy number has been documented in some types of cancers, such as ovarian epithelial tumor, glioblastoma, and invasive breast carcinoma [The cBioPortal for Cancer Genomics (http://cbioportal.org)]. However, the pathophysiological roles of VIPR2 in cancer remain largely unknown.

VIPR2 is strongly coupled through Gαs protein to adenylyl cyclase. VIP stimulation leads to cAMP production and subsequent activation of protein kinase A (PKA) and exchange protein directly activated by cAMP (EPAC) (15, 28, 29). Both of these activated proteins are involved in activation of the downstream effector extracellular signal-regulated kinase (ERK). Other signaling pathways activated by VIPR2 have also been reported. For example, VIPR2 couples with Gαi and Gαq proteins, which regulate signaling molecules as diverse as SRC, PI3K, phospholipase C, and protein kinase C (15, 30–32). VIP caused a concentration-dependent increase in both cAMP and [^3^H]inositol phosphate production in GH3 rat pituitary tumor cells that natively express VIPR2 but not other VIP-responsive receptors. PACAP also induced cAMP and [^3^H]inositol phosphate production in COS7 cells transiently expressing rat VIPR2 (32). Additionally, PACAP promoted the phosphorylation of AKT, an effector molecule of PI(3,4,5)P_3_, in the MCF-7 breast cancer cell line that expresses VIPR1 and VIPR2 (33), suggesting that activation of VIP receptor-mediated signaling may increase PI(3,4,5)P_3_ and promote cancer cell migration. Overexpression of VIPR1 has been shown to inhibit migration and invasion of human lung adenocarcinoma H1299 cells (25). However, a role for VIPR2 signaling in cancer cell migration has not been completely elucidated.

In the present study, we investigated the potential roles and mechanisms of VIPR2 in controlling the PI3K/PI(3,4,5)P_3_ pathway and influencing cancer cell migration.

## Materials and methods

### Plasmids, siRNAs, and synthetic peptide

The pCMV6-AN-Myc-DDK vector (PS100016) and a negative control siRNA (S10C-0600) were purchased from Cosmo Bio (Tokyo, Japan). The pEGFP-N2 vector and pmCherry-N1 vector were purchased from Takara Clontech (Kyoto, Japan). The pCMV6-VIPR2-Myc-DDK plasmid was constructed from the pCMV6-AN-Myc-DDK vector and VIPR2 cDNA. The VIPR2-Myc region from the resultant plasmid was cloned into the pEGFP-N2 vector, and the VIPR2-Myc-DDK region was cloned into pmCherry-N1 vector. The AKT-PH-EGFP vector was described previously (34). Human *VIPR2*-siRNAs (si1, 3024813653-000080 and -000090; si2, 3024813653-000020 and -000030; si3, 3024813653-000050 and -000060) were purchased from Sigma-Aldrich (St. Louis, MO, USA). A VIPR2-selective antagonistic peptide KS-133 was synthesized at SCRUM Inc. (Tokyo, Japan) as previously reported (35).

### Antibodies

Anti-ARP3 (#4738), anti-GAPDH (#2118), anti-PI3-kinase p110α (#4249), anti-PI3-kinase p110β (#3011), anti-PI3-kinase p110γ (#5405), anti-PI3 Kinase Class III (#3358), anti-WAVE2 (#3659), anti-pan AKT (#4691), anti-phospho-AKT (Thr308; #2965), HRP-conjugated anti-rabbit IgG (#7074) and HRP-conjugated anti-mouse IgG (#7076) were purchased from Cell Signaling Technology (Beverly, MA, USA). Anti-mCherry (ab167453) and anti-VIPR2 (ab183334) were purchased from Abcam (Cambridge, United Kingdom). Anti-GFP (sc-9996) was purchased from Santa Cruz Biotechnology (Dallas, TX, USA). Anti-GAPDH (60004-1-Ig) was purchased from Proteintech (Chicago, IL, USA). Alexa Fluor 594 anti-rabbit IgG (A-11012), Alexa Fluor 405 anti-mouse IgG (A-31553) and Alexa Fluor 555 anti-mouse IgG (A-21422) were purchased from Invitrogen (Carlsbad, CA, USA).

#### Cells, cell transfection and production of stable cell lines

The MDA-MB-231 cell line was purchased from JCRB Cell Bank (JCRB; Tokyo, Japan). The development of MCF-7 cells stably expressing EGFP was described previously (34). Cells were cultured under conventional growth conditions.

Cells were transfected with siRNAs or plasmids using Lipofectamine 3000 (Invitrogen) in accordance with the manufacturer’s instructions.

To establish stable cell lines, MDA-MB-231 and MCF-7 cells were transfected with an expression vector encoding VIPR2-EGFP or a control EGFP vector and cultured in the presence of 1 mg/ml G418 (Nacalai Tesque, Kyoto, Japan) for 14 days. Stably EGFP-expressing colonies were selected. HeLa cells stably expressing VIPR2-mCherry were produced using similar methods. LLC-PK1 cells stably expressing EGFP-actin were kindly supplied by Dr. Keiju Kamijo (Tohoku Medical and Pharmaceutical University).

#### Reverse transcription-PCR analysis

Total RNA was isolated from cells using a RNeasy mini kit (Qiagen, Hamburg, German). RNA (5 μg) was then subjected to reverse transcription reactions using a cDNA synthesis kit (Takara Bio, Tokyo, Japan). Human *ADCYAP1R1* (*PAC1*), *VIPR1*, *VIPR2* and *GAPDH* sequences were amplified by PCR using the following primers: 5′-TTCAATGATTCCTCTCCAGGCTG-3′ and 5′-GGCCTTCACTGACAGGTAGTAATA-3′ for *PAC1*, 5′-TGTGAAGACCGGCTACACCA -3′ and 5′-TCCACCAGCAGCCAGAAGAA-3′ for *VIPR1*, 5′- CATGGACTGCGGCCAGG-3′ and 5′-GAACGACCCGAGGCACAG-3′ for *VIPR2*, and 5′-ACCACAGTCCATGCCATCAC-3′ and 5′- TCCACCACCCTGTTGCTGTA-3′ for *GAPDH.* The PCR products were distinguished by agarose gel electrophoresis.

#### Thin-layer chromatography

For the assessment of PI3K activity *in vitro*, cells were lysed and the lysates were incubated with BODIPY FL-PtdIns(4,5)P_2_ (C-45F6a; Echelon, San Jose, CA, USA) in the manufacturer’s buffer containing DTT and ATP for 60 min at 37°C. The lipids were extracted by CHCl_3_/MeOH (v/v=2:1) using the methods of Bligh and Dyer (36). Samples were applied to thin-layer chromatography plates and developed in CHCl_3_/acetone/MeOH/AcOH/water (v/v/v/v/v = 40:15:13:12:7) using the methods of Huang et al. (37). The fluorescent lipids were visualized by UV irradiation using the ChemiDoc XRS system (Bio-Rad, Hercules, CA, USA).

#### Measurement of PI(3,4,5)P_3_ levels at the plasma membrane

The AKT-PH-EGFP vector was co-expressed in MDA-MB-231 cells transfected with *VIPR2* siRNA1 or *VIPR2*-mCherry, and the cells were treated with 100 nM of VIP for 10 min. The EGFP intensities on the plasma membrane were measured and normalized by the intensity of whole cells.

#### Immunofluorescence, western blotting, and pull-down assay

Immunofluorescence and western blotting were carried out following previously described methods (38, 39). The co-localization analysis of fluorescence microscopy images was performed using Image-Pro premier ver.9.4.

Pull-down assays with GFP-tagged protein complexes were performed using GFP-Trap_A beads (ChromoTek, Planegg-Martinsried, Germany) in accordance with the manufacturer’s instructions. LLC-PK1 cells stably expressing EGFP-actin were lysed with the manufacturer’s buffer containing protease inhibitors and then centrifuged at 15,000 × g for 30 min at 4°C. The resulting supernatant was incubated with GFP-Trap_A beads overnight at 4°C with gentle rotation. After gentle centrifugation, the precipitates in the pull-down fraction were boiled in SDS sample buffer, separated by SDS-PAGE, and analyzed by western blotting using anti-WAVE2 and anti-ARP3 antibodies.

#### Random migration assay, transwell migration assay and scratch wound closure assay

MCF-7 cells (1.5 × 10^4^) were seeded in 35 mm culture dishes in serum-free medium, cultured until attached, and stimulated with 100 nM VIP (Cayman Chemical Company, Ann Arbor, MI, USA). For the migration assay, cells were monitored every 1 h for 12 h on a thermo plate (Tokai Hit, Shizuoka, Japan) at 37°C by live-cell imaging (BZ-X800; Keyence, Osaka, Japan). Tracking analysis of cells was performed using Image-Pro premier ver.9.4 (Media Cybernetics, Rockville, MD, USA).

Cell migration was also examined using a Boyden chamber following previously described methods (40). Briefly, the lower chamber was filled with 600 μL of 200 nM VIP in serum-free culture medium. MDA-MB-231 cells stably expressing EGFP (1 × 10^4^ cells) were suspended in 100 μL of serum-free culture medium and transferred to the cell culture insert. The cells were allowed to migrate for 30 or 48 h at 37°C. Non-migrating cells on the upper surface of the membrane were scraped off, and migratory cells attached to the lower surface were fixed by 4% paraformaldehyde and counted under a fluorescence microscope.

For a scratch assay, MDA-MB-231 cells transfected with *VIPR2* siRNA1 or *VIPR2*-EGFP were seeded into 12-well tissue culture dishes coated with fibronectin (50 μg/mL) and cultured in medium containing 10% FBS to nearly confluent cell monolayers. Then, a linear wound was generated in the monolayer with a sterile plastic pipette tip. Any cellular debris was removed by washing with serum-free medium. The serum-free culture medium with 100 nM VIP and 50 mM Hepes (pH 7.4) was added to each well, and cells were monitored for 24 h on a thermo plate at 37°C by live-cell imaging on a BZ-X800 microscope.

#### Kymograph analysis of the extension of cell leading edge

To observe the extension of the leading edge of migrating cells, cells were seeded on μ-dishes (Ibidi, Martinsried, Germany), cultured until confluent, wounded with a pipet tip, and stimulated with 100 nM VIP. The cells were recorded every 2 min for 60 min by live-cell imaging on a BZ-X800 microscope. Kymography analysis of the leading edge was performed using the MultipleKymograph plug-in of ImageJ 1.53a (National Institutes of Health; developed by J. Rietdorf and A. Seitz).

#### Statistical analysis

GraphPad Prism was used for the statistical analyses. A non-parametric Mann–Whitney *U* test (for two groups) and Kruskal–Wallis test (for more than two groups) followed by Dunn’s multiple comparison test were used. A p*-*value of less than 0.05 was considered statistically significant.

## Results

### VIP–VIPR2 signaling promotes the PI3K/PI(3,4,5)P_3_ pathway in cancer cells

To investigate whether VIP–VIPR2 signaling is involved in PI3K/PI(3,4,5)P_3_ pathway-mediated cell migration, we examined the effects of VIP on the phosphorylation of AKT in cancer cells. PI3Ks are activated by the stimulation of several kinds of chemokines. Activated PI3K produces PI(3,4,5)P_3_ and recruits AKT to the plasma membrane by binding between AKT PH domain and PI(3,4,5)P_3_, which induces AKT Thr308 phosphorylation, followed by AKT Ser473 phosphorylation by mammalian target of rapamycin 2 (mTORC2) (41, 42). All cancer cell lines examined in this study expressed *VIPR2* mRNA (**Figure S1**), and VIPR2 protein was detected in MCF-7 and MDA-MB-231 cells (human breast cancer cell lines) and HeLa cells (human cervical cancer cell line) (**Figure S2A**).

**Figure 1:**
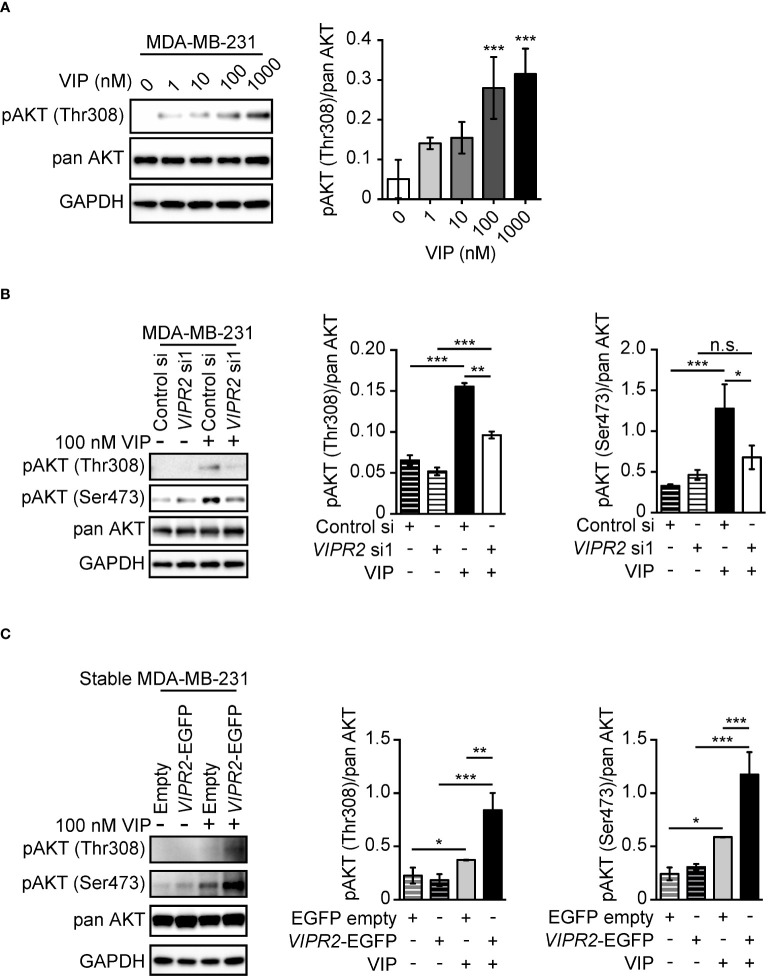
VIP–VIPR2 signaling regulates the phosphorylation of AKT in MDA-MB-231 cells. **(A)** MDA-MB-231 cells were starved for 3 h and then stimulated with the indicated doses of VIP for 10 min. Treated cells were lysed and evaluated by western blotting using specific antibodies for the indicated proteins. A set of representative images is shown. The graph shows the density of pAKT (Thr308) normalized against the corresponding density of the respective pan AKT. The data are presented as means ± SD (n = 3); ***p < 0.001 (versus 0 nM VIP; Kruskal–Wallis test followed by Dunn’s multiple comparison test). **(B)** MDA-MB-231 cells transfected with the indicated siRNAs were stimulated with 100 nM VIP for 10 min. Cell lysates were analyzed by western blotting to detect the indicated proteins. The graphs show the density of pAKT (Thr308 or Ser473) normalized against the corresponding density of the respective pan-antibody bands. The data are presented as means ± SD (n = 3); *p < 0.05, **p < 0.01, ***p < 0.001 between the indicated groups (Kruskal–Wallis test followed by Dunn’s multiple comparison test). n.s. indicates not statistically significant. **(C)** MDA-MB-231 cells stably expressing EGFP (Empty) or VIPR2-EGFP were stimulated with VIP for 10 min. Cell lysates were analyzed by western blotting using the indicated specific antibodies. The graphs show the density of pAKT (Thr308 or Ser473) normalized against the corresponding density of the respective pan AKT. The data are presented as means ± SD (n = 3); *p < 0.05, **p < 0.01, ***p < 0.001 between the indicated groups (Kruskal–Wallis test followed by Dunn’s multiple comparison test) ns, not significant..

We observed increased phosphorylation of AKT at Thr308 in MDA-MB-231 cells treated with 100 nM and 1000 nM VIP at 10 min (**Figure 1A**). Silencing of *VIPR2* in MDA-MB-231 cells (**Figure S2A**) markedly inhibited the phosphorylation of AKT (**Figure 1B**). Together these results indicate that VIPR2 couples to the AKT pathway in these cells.

We next established several cancer cell lines stably expressing exogenous VIPR2 (**Figures S2A, B**). In the presence of 100 nM VIP, phosphorylated AKT at Thr308 and Ser473 was markedly increased in cells stably expressing VIPR2 compared with that of empty vector-expressing MDA-MB-231 cells (**Figure 1C**).

We also observed increased phosphorylation of AKT at Ser473 in MCF-7 cells treated with 100 nM VIP, with the maximum effect observed at 5 to 10 min (**Figures S3A, B**). Silencing of *VIPR2* in MCF-7 cells (**Figure S2A**) markedly inhibited the phosphorylation of AKT (**Figure S3B**). These results suggest that 100 nM VIP may stimulate the PI3K/PI(3,4,5)P_3_ pathway through VIPR2. VIP-VIPR2 signaling also strongly activates ERK through a Gαs protein–dependent pathway (15). ERK1/2 phosphorylation in *VIPR2*-silenced MCF-7 cells was similar to that of control cells (**Figure S3B**), suggesting that other receptors binding to VIP compensated for the deficiency of VIPR2 for ERK1/2 phosphorylation.

### VIP–VIPR2 signaling regulates PI3K activity and consequently controls cell membrane PI(3,4,5)P_3_ levels

**Figure 2:**
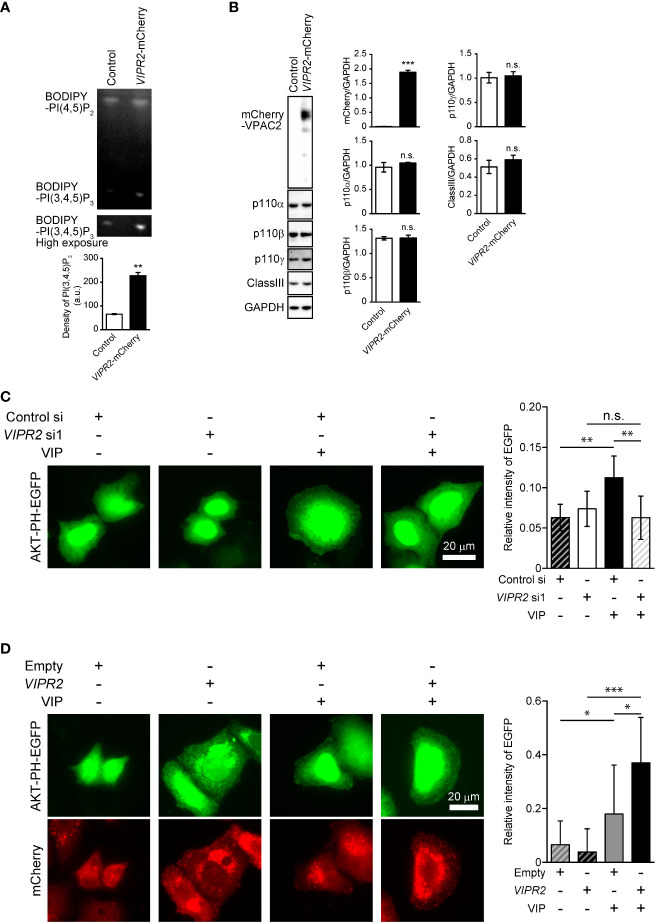
VIP–VIPR2 signaling regulates the activity of PI3K and subsequent PI(3,4,5)P_3_ level. **(A)** HeLa cells stably expressing mCherry-tagged VIPR2 or control vector were used for experiments. The cell lysates were incubated with BODIPY FL-PI(4,5)P_2_ for 1 h at 37°C, and the lipids extracted from cells were distinguished by thin layer chromatography. The lower panel was obtained using a high sensitivity mode (high exposure). The intensities of BODIPY bands in the top panel were quantified using ImageJ software, and the data are presented in the graph as means ± SD (n = 3). **p < 0.01 (Mann–Whitney U test). **(B)** Cell lysates were analyzed by western blotting to detect the indicated proteins. The density of mCherry-VIPR2, p110α, p110β, p110γ and classIII were normalized against that of GAPDH. The data are presented as means ± SD (n = 3). ***p < 0.001 (Mann–Whitney’s U test). n.s.: not significant. **(C)** MDA-MB-231 cells transfected with EGFP-tagged AKT-PH plasmid and either *VIPR2* siRNA1 or control siRNA were stimulated with 100 nM VIP for 10 min and then fixed; EGFP was detected by fluorescent microscopy. A set of representative images from 3 independent experiments is shown. The plasma membrane was defined as the region 3 μm from the cell periphery. Relative fluorescence intensity of AKT PH-EGFP (plasma membrane/whole cell) was calculated. The data are presented in the graph as means ± SD [n = 30 (30 cells in each group)]. **p < 0.01, between the indicated groups (Kruskal–Wallis test followed by Dunn’s multiple comparison test). n.s. indicates not statistically significant. **(D)** MDA-MB-231 cells transfected with EGFP-tagged AKT-PH plasmid and either empty vector or *VIPR2*-mCherry were stimulated with 100 nM VIP for 10 min and then fixed; EGFP and mCherry were detected by fluorescent microscopy. A set of representative images from 3 independent experiments is shown. Relative fluorescence intensity of AKT PH-EGFP (plasma membrane/whole cell) was calculated. The data are presented in the graph as means ± SD [n = 20 (20 cells in each group)]. *p < 0.05, ***p < 0.001, between the indicated groups (Kruskal–Wallis test followed by Dunn’s multiple comparison test) ns, not significant.

To investigate the involvement of VIPR2 in the PI3K/PI(3,4,5)P_3_ pathway, we analyzed phospholipid metabolism in whole cell lysates from VIP-stimulated VIPR2-mCherry-expressing HeLa cells (**Figures S2A, B**) using an *in vitro* PI3K activity assay. HeLa cells have no expression of *VIPR1* and *PAC1* mRNA (Figure S1), suggesting that HeLa cells are suitable for analysis of VIP–VIPR2 signaling. The metabolism of PI(4,5)P_2_ to PI(3,4,5)P_3_ was increased approximately four-fold in VIPR2-overexpressing HeLa cell lysates compared with that in controls (**Figure 2A**), but the expression of the catalytic subunit of PI3K in whole cell lysates showed no changes in the control or exogenous VIPR2-expressing cells (**Figure 2B**).

We next examined changes in PI(3,4,5)P_3_ on the plasma membrane after VIP stimulation using *VIPR2*-silenced MDA-MB-231 cells that overexpressed an EGFP-tagged Akt PH domain (Akt PH-EGFP), a probe for PI(3,4,5)P_3_. Accumulation of AKT PH-EGFP signals on the plasma membrane of control siRNA-transfected MDA-MB-231 cells was observed at 10 min after VIP stimulation (**Figure 2C**). However, the signals were decreased in *VIPR2*-silenced cells (**Figure 2C**) and markedly increased in cells stably expressing VIPR2 (**Figure 2D**). Together, these data suggest that VIP–VIPR2 signaling regulates PI3K activity and the subsequent production of PI(3,4,5)P_3_ in the plasma membrane.

### VIPR2 is involved in cell migration of MDA-MB-231 and MCF-7 breast cancer cells

**Figure 3:**
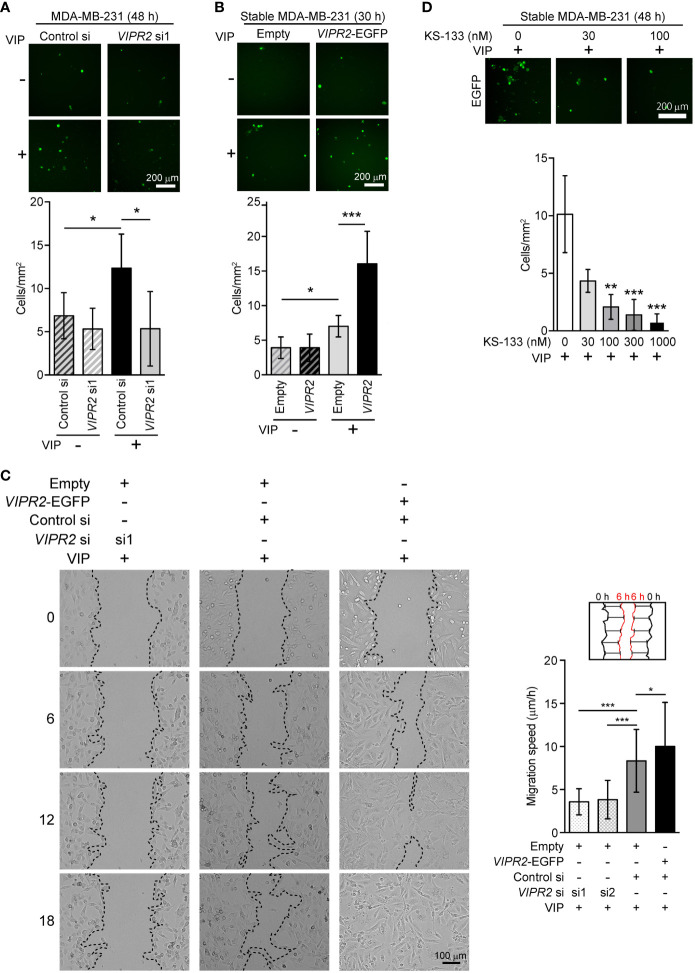
Cell motility in *VIPR2*-silenced and VIPR2-expressing MDA-MB-231 cells. (A and B) Migration of EGFP-expressing MDA-MB-231 cells transfected with either control siRNA or *VIPR2* siRNA1 **(A)** and that of MDA-MB-231 cells stably transfected with either *VIPR2-EGFP* or *EGFP* plasmid **(B)** was measured using a transwell migration assay. VIP (200 nM) was added to the lower chamber of the vessel. Cells were added to the upper chamber and incubated for 48 h **(A)** or 30 h **(B)** at 37°C. Quantification of migrating cells is shown in graphs. The data are presented as means ± SD [n = 9 (3 independent experiments; 3 areas were randomly selected in each group at each experiment)]. *p < 0.05, ***p < 0.001 between the indicated groups (Kruskal–Wallis test followed by Dunn’s multiple comparison test). **(C)** Wound healing *in vitro* in the scratch assay using confluent monolayer of *VIPR2*-silenced or VIPR2-expressing MDA-MB-231 cells was monitored for 18 h after 100 nM VIP stimulation. The dotted line indicates top position of the cell population. The distance migrated during 6 h was measured at 10 randomly selected points (*see* schematic diagram), and the average speed is shown in graphs. The data are presented as means ± SD [n = 90 (3 independent experiments; 10 points in 3 areas randomly selected in each group at each experiment)]. *p < 0.05, ***p < 0.001 between the indicated groups (Kruskal–Wallis test followed by Dunn’s multiple comparison test). **(D)** Migration of MDA-MB-231 cells stably transfected with *VIPR2-EGFP* was measured using a transwell migration assay. VIP (200 nM) was added to the lower chamber of the vessel. The indicated doses of KS-133 were added to both chambers. Cells were added to the upper chamber and incubated for 48 h at 37°C. Quantification of migrating cells is shown in graph. The data are presented as means ± SD [n = 9 (3 independent experiments; 3 areas were randomly selected in each group at each experiment)]. **p < 0.01, ***p < 0.001 between the indicated groups (Kruskal–Wallis test followed by Dunn’s multiple comparison test).

To investigate whether VIPR2 expression affects cancer cell motility, we examined the cell migration of MDA-MB-231 cells. We used a transwell chamber assay (**Figures 3A, B**) and scratch wound closure assay (**Figure 3C**) to evaluate the migration of MDA-MB-231 cells. The migration of *VIPR2* siRNA-transfected cells stimulated by VIP was reduced compared with that of control siRNA-transfected cells (**Figures 3A, C**); in contrast, the motility of MDA-MB-231 cells was promoted by the expression of VIPR2-EGFP (**Figures 3B, C**). A random migration assay also revealed that MCF-7 cells transfected with *VIPR2* siRNA moved shorter distances (**Figures S4A, B**) with a slower migration speed (**Figure S3C**) compared with control siRNA-transfected cells. We next generated MCF-7 cells stably expressing VIPR2-EGFP (**Figures S2A, B**) and performed random migration assays. In line with the effects of *VIPR2*-silencing, the migration speed was higher in VIPR2-EGFP-expressing MCF-7 cells than that in EGFP-expressing cells (**Figure S4C**). Notably, treatment with KS-133, a potent and selective VIPR2 antagonist (35), dose-dependently inhibited VIP-induced cell migration in VIPR2-overexpressing MDA-MB-231 cells (**Figure 3D**). These results suggest that VIPR2 mediates cell migration induced by VIP.

### VIPR2-mediated upregulation of PI3K is essential for VIP-induced cell migration

**Figure 4:**
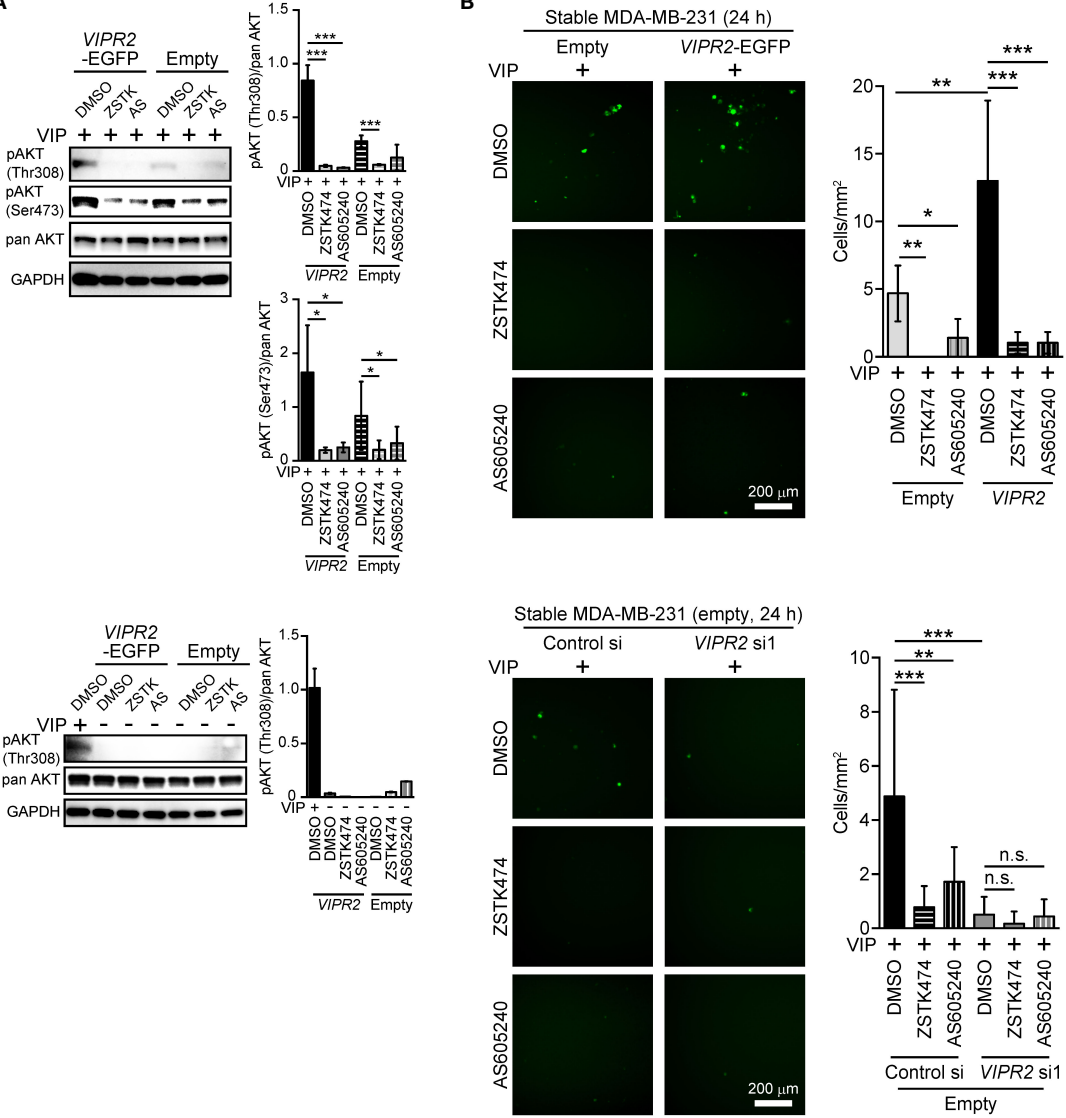
PI3Kγ inhibitor attenuates VIPR2-mediated cell migration. **(A)** MDA-MB-231 cells stably expressing the indicated proteins were stimulated with 100 nM VIP for 10 min (upper panels) or 0 min (lower panels) in the presence of the indicated drugs (10 μM ZSTK, 6 μM AS605240, 0.2% DMSO). Cell lysates were analyzed by western blotting to detect the indicated proteins. The graphs display the density of pAKT (Thr308 or Ser473) normalized against the corresponding density of the respective pan AKT. The data are presented as means ± SD (n = 3); ***p < 0.001 between the indicated bars (Kruskal–Wallis test followed by Dunn’s multiple comparison test). **(B)** Migration of MDA-MB-231 cells transfected with either *VIPR2-EGFP* or *EGFP* plasmid (upper panels) or *EGFP* plasmid together with the indicated siRNAs (lower panels) was measured using a transwell migration assay. VIP (200 nM) and the indicated drugs (10 μM ZSTK, 6 μM AS605240, 0.2% DMSO) were added to the lower chamber. Cells were added to the upper chamber with the indicated drugs and incubated for 24 h at 37°C. Quantification of the migrating cells is shown in the graph. The data are presented as means ± SD [n = 9 (3 independent experiments; 3 areas were randomly selected in each group at each experiment)]. *p < 0.05, **p < 0.01, ***p < 0.001 between the indicated groups (Kruskal–Wallis test followed by Dunn’s multiple comparison test). n.s. indicates not statistically significant.

To investigate the role of increased PI3K activity in VIP-induced VIPR2-mediated cell migration, we applied PI3K inhibitors to VIP-stimulated MDA-MB-231 cells. Western blot analysis revealed that either 10 μM of ZSTK474 (a pan-PI3K class I inhibitor) or 6 μM of AS605240 (a PI3Kγ inhibitor) treatment markedly suppressed the VIP-induced phosphorylation of AKT in both EGFP-expressing MDA-MB-231 cells and MDA-MB-231 cells stably expressing VIPR2-EGFP (**Figure 4A**).

We then performed transwell chamber migration assays with MDA-MB-231 cells treated with the inhibitors. Both ZSTK474 and AS605240 significantly decreased cell migration of VIPR2-EGFP-expressing MDA-MB-231 cells to the level observed in EGFP-expressing MDA-MB-231 cells (**Figure 4B**, upper panels). In contrast, cell migration was almost completely suppressed by transfection with *VIPR2* siRNA, and *VIPR2*-silenced cells were no longer affected by the treatment with ZSTK474 or AS605240 (**Figure 4B**, lower panels). These results indicate that VIP–VIPR2 signaling thus regulates PI3Kγ-mediated cell migration, most likely *via* increased PI3Kγ activity.

### VIPR2 regulates PI(3,4,5)P_3_-mediated downstream signaling

**Figure 5:**
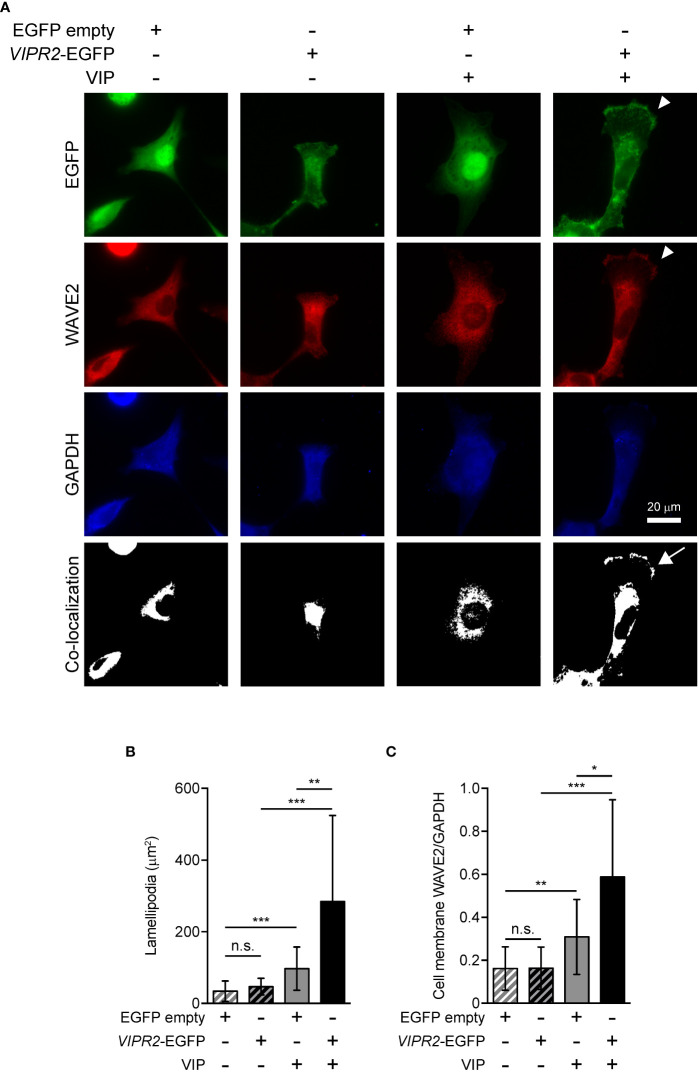
VIPR2 overexpression promotes accumulation of WAVE2 in the cell membrane. **(A)** MDA-MB-231 cells transfected with *VIPR2-EGFP* or *EGFP* were stimulated with (+) or without (−) 100 nM VIP for 10 min. Cells were evaluated by immunocytochemistry using the antibodies against the indicated proteins. Arrowheads indicate lamellipodia membrane. The white color regions show the co-localization between EGFP and WAVE2. Arrow indicates co-localization on the lamellipodia membrane. Similar images were obtained from three independent experiments, and representative images are shown. **(B)** Bar graph shows quantification of the area of the lamellipodia membrane. **(C)** WAVE2 intensity on the cell membrane was calculated from data shown in **(A)** and normalized by the intensity of GAPDH (whole cell). The data are presented as means ± SD [n = 30 (30 cells in each condition)]. *p < 0.05, **p < 0.01, ***p < 0.001, between the indicated groups (Kruskal–Wallis test followed by Dunn’s multiple comparison test). n.s. indicates not statistically significant **(B, C)**.

WAVE2 binds to PI(3,4,5)P_3_ on the plasma membrane and then interacts with the ARP2/3 complex, which leads to actin polymerization (7). To investigate whether upregulation of PI(3,4,5)P_3_ on the plasma membrane in VIPR2-overexpressing MDA-MB-231 cells affect WAVE2 localization to the plasma membrane, we performed immunocytochemistry with an anti-WAVE2 antibody (**Figure 5A**). In the absence of VIP stimulation, VIPR2-EGFP was distributed across most of the plasma membrane; upon treatment of cells with VIP, VIPR2-EGFP localized to the plasma membrane in lamellipodia (**Figure 5A**). The area of lamellipodia in VIP-induced cell extension was increased in VIPR2-overexpressing MDA-MB-231 cells compared with that in control cells at 10 min after stimulation (**Figure 5B**). Without VIP stimulation, the WAVE2 signal on the plasma membrane was hardly detected in either cell line. In contrast, at 10 min after VIP stimulation, WAVE2 accumulated at the plasma membrane (**Figure 5A**), and the signals on the plasma membrane were stronger in VIPR2-overexpressing MDA-MB-231 cells than that in EGFP vector-transfected MDA-MB-231 cells (**Figure 5C**). The WAVE2 signal was co-localized with VIPR2-EGFP signals at the plasma membrane in lamellipodia (**Figure 5A**).

**Figure 6:**
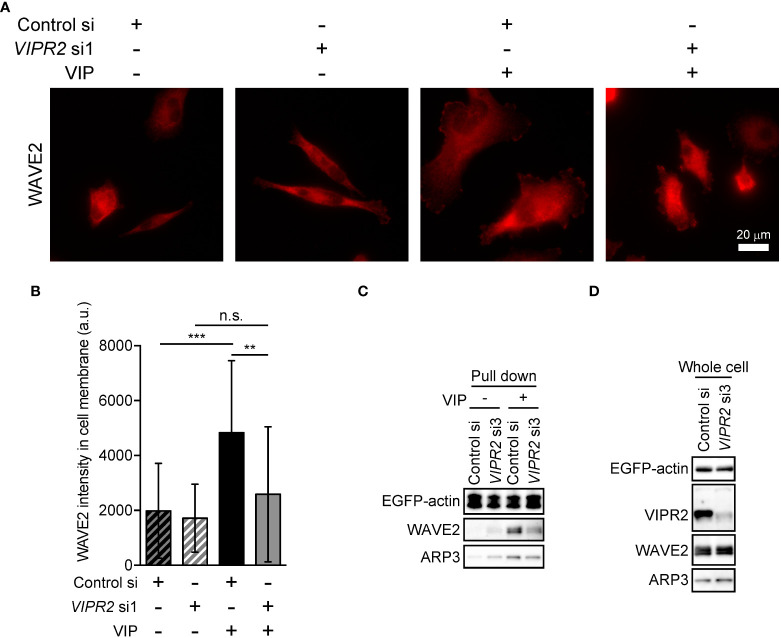
VIPR2 mediates the interaction between WAVE2, ARP and actin. **(A)** MDA-MB-231 cells transfected with either control siRNA or *VIPR2* siRNA1 were stimulated with (+) or without (−) 100 nM VIP for 10 min. Cells were evaluated by immunocytochemistry using an anti-WAVE2 antibody. Representative images are shown. **(B)** Bar graph shows quantification of the fluorescence intensity of WAVE2 on the plasma membrane. The data are presented as means ± SD [VIP **(-)** and control si, 76 cells; VIP **(-)** and *VIPR2* si1, 104 cells; VIP (+) and control si, 66 cells; VIP (+) and *VIPR2* si1, 130 cells in total]. **p < 0.01, ***p < 0.001, between the indicated groups (Kruskal–Wallis test followed by Dunn’s multiple comparison test). n.s. indicates not statistically significant. **(C, D)** Pull-down assay using GFP-Trap_A beads was performed to evaluate the amount of WAVE2 and ARP3 bound to EGFP-actin. LLC-PK1 cells stably expressing EGFP-actin were transfected with *VIPR2* siRNA3; the pull-down fraction was collected using GFP-Trap_A beads and centrifugation. Western blotting of the obtained fractions (pull down, C) and whole cell lysates **(D)** was performed using the specific antibodies for the indicated proteins. EGFP-actin was detected by a GFP antibody. Similar data were obtained from three independent experiments, and representative images are shown.

To further verify the involvement of VIPR2 in WAVE2 translocation to the plasma membrane, we silenced *VIPR2* and examined the localization of WAVE2 (**Figure 6**). In response to VIP stimulation, MDA-MB-231 cells transfected with *VIPR2* siRNA showed lower levels of WAVE2 signal at the plasma membrane than those in control cells (**Figures 6A, B**).

Activated WAVE2 binds to the ARP2/3 complex and actin (7). We thus examined the interaction between WAVE2, ARP3 and EGFP-actin *in vitro* by pull-down assays using cell lysates of LLC-PK1 pig kidney epithelial cells stably expressing EGFP-actin (provided by Dr. Keiju Kamijo). Both endogenous WAVE2 and ARP3 co-precipitated with EGFP-actin to a lesser degree in *VIPR2*-silenced cells treated with VIP compared with that in control siRNA-transfected LLC-PK1 cells treated with VIP (**Figure 6C**). However, *VIPR2*-silencing had no effects on the total expression levels of WAVE2 and ARP3 (**Figure 6D**).

### VIPR2 participates in lamellipodium extension in MDA-MB-231 cells

**Figure 7:**
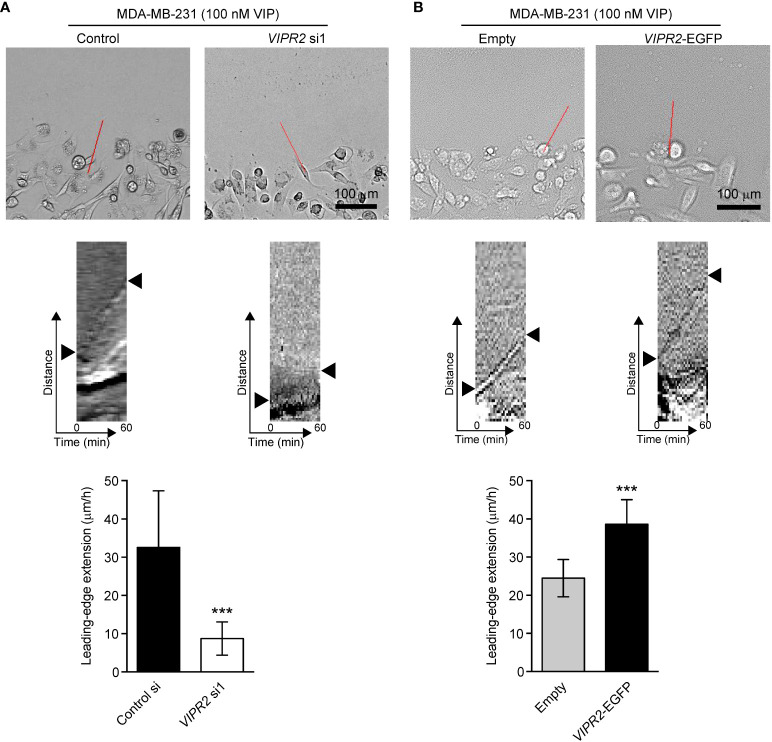
VIPR2 regulates lamellipodium extension in MDA-MB-231 cells. **(**A, B) Extension of the leading-edge (arrowheads in the middle panels) in MDA-MB-231 cells transfected with either control siRNA or *VIPR2* siRNA1 **(A)** and MDA-MB-231 cells stably expressing either EGFP or VIPR2-EGFP **(B)** was monitored during wound healing. The images were acquired at 2-min intervals for 60 min after 100 nM VIP stimulation. Top panels show images at time 0. The kymographs (middle panels) were analyzed at the red lines for 0–60 min. Similar results were obtained from three independent experiments and representative images are shown. Quantification of the extended leading-edge for 1 h is shown in graphs (bottom panels). The data are presented as means ± SD [n = 15 (3 independent experiments; 5 cells were randomly selected in each group at each time)]. ***p < 0.001 (Mann–Whitney’s U test).

WAVE2-mediated actin nucleation and subsequent conformation of actin filament drives lamellipodium formation and extension (7). We next performed a scratch wound closure assay to investigate the effect of VIPR2 on lamellipodium extension at the leading edge of migrating cells and analyzed lamella dynamics at the leading edge by kymography (**Figure 7**). VIP stimulation-induced lamellipodium extension was attenuated in *VIPR2*-silenced MDA-MB-231 cells compared with that in control cells (**Figure 7A**). In contrast, the lamellipodium extension extended further in MDA-MB-231 cells transfected with VIPR2-EGFP plasmid compared with the distance in empty vector-transfected cells (**Figure 7B**).

## Discussion

**Figure 8:**
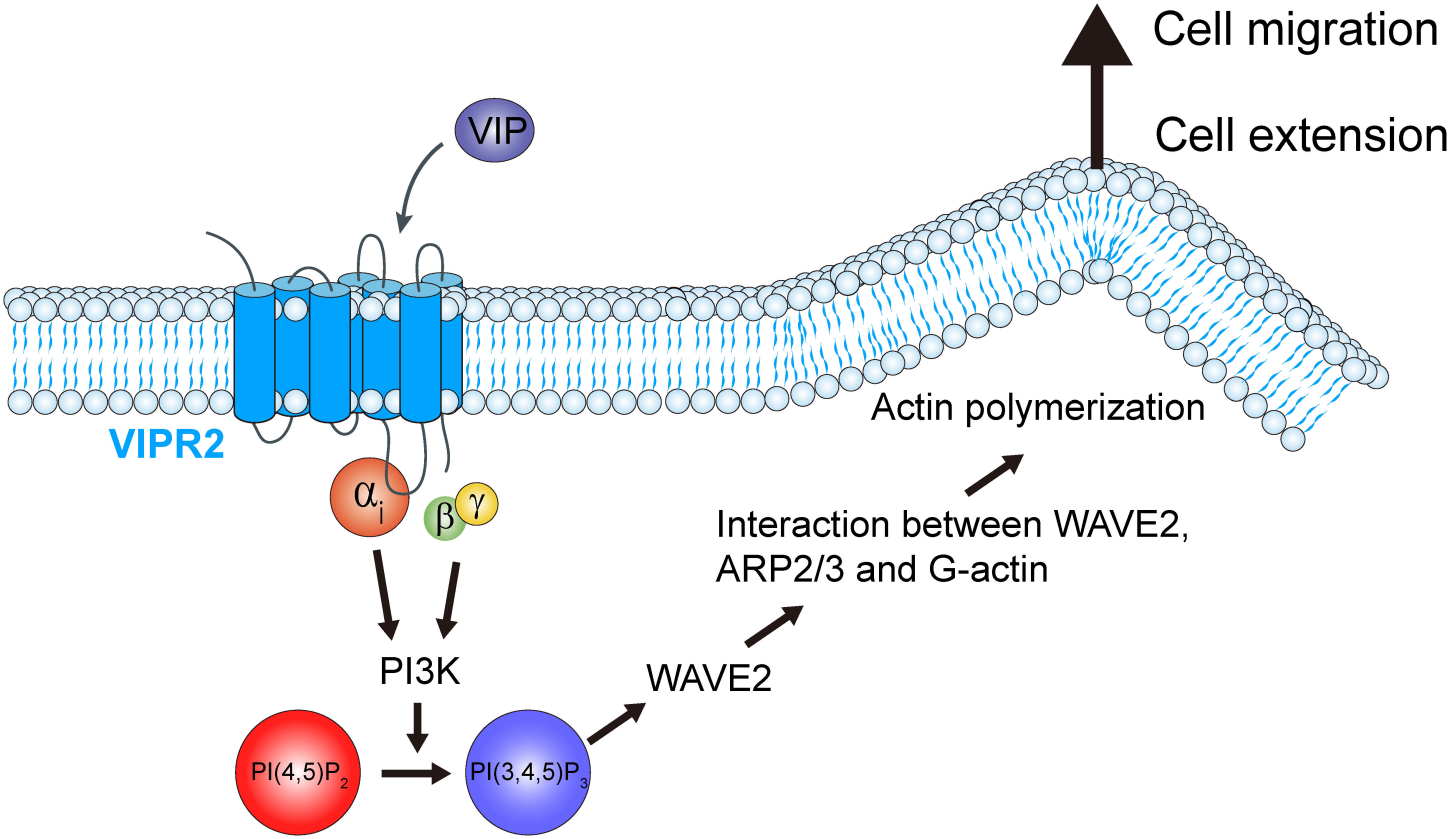
Schematic representation of the role of VIPR2 in breast cancer cell migration. Increased *VIPR2* mRNA expression and/or *VIPR2* gene copy number has been found in invasive breast carcinoma. VIPR2 couples with Gαs, Gαi and Gαq proteins. Gαi-coupled VIPR2 and its Gβγ subunits regulate PI3Ks activity. PI3Ks convert phosphatidylinositol 4,5-bisphosphate [PI(4,5)P_2_] into phosphatidylinositol 3,4,5-trisphosphate [PI(3,4,5)P_3_]. The synthesized PI(3,4,5)P_3_ promotes the translocation of WASP family verprolin homologous protein 2 (WAVE2) to the plasma membrane. WAVE2 drives lamellipodium formation by enhancing actin nucleation *via* the actin-related protein 2 and 3 (ARP2/3) complex, resulting in promotion of breast cancer cell migration.

The results of this study revealed that VIP–VIPR2 signaling participates in a PI3K-induced PI(3,4,5)P_3_ production-mediated signaling pathway involved in cell migration. VIPR2 strongly couples with Gαs protein and mediates PKA and its downstream signaling pathway; in some cases, VIPR2 also couples with Gαi and Gαq proteins (15, 30–32). Signaling through Gαi protein promotes the PI3K/AKT pathway, which is associated with increased PI(3,4,5)P_3_. Indeed, we found that VIPR2 overexpression leads to further activation of PI3K and additional increase of PI(3,4,5)P_3_ followed by WAVE2-dependent cell extension and cancer cell migration (**Figure 8**). These results contribute to the understanding of the PI3K/PI(3,4,5)P_3_ signaling pathway and suggest VIPR2 as a new potential therapeutic target against cancer.

Constitutive activation of the PI3K/AKT signaling cascade is common in cancer. Aberrant activation of PI3K/AKT signaling in cancer typically occurs from mutations in genes encoding receptor tyrosine kinases, PI3K, phosphatase and tensin homologue (PTEN), or AKT. Moreover, the reduced expression or ablation of PTEN, a PI(3,4,5)P_3_ phosphatase, results in malignant transformation (11, 12, 43). The deregulation of PI3K activity (e.g., by H1047R and E542K in p110α) is associated with high malignancy and an increased resistance to chemotherapeutic and radiation therapies in breast cancer. The elucidation of the roles of VIPR2 in PI3K activity revealed in this study and the increased *VIPR2* gene expression in some cancers suggest the possibility that VIPR2 may play a part in cancer development.

Our experiments with PI3K inhibitors revealed that phosphorylated AKT level in VIP-stimulated VIPR2-EGFP-expressing cells was almost completely eliminated by treatment with a PI3Kγ-specific inhibitor AS605240. AS605240 also suppressed the VIP-induced cell migration in VIPR2-EGFP-expressing cells to the level observed in the treatment with a pan-PI3K class I inhibitor ZSTK474. Moreover, in *VIPR2*-silenced cells, treatment with either AS605240 or ZSTK474 had no further effects on cell migration. Together, these results suggest that PI3Kγ plays a key role as a downstream molecule of VIPR2 signaling in regulating cell migration. Since PI3Kγ is activated by Gβγ (5), VIP–VIPR2 signaling likely activates PI3Kγ in a Gβγ-dependent manner, leading to increased cell migration.

VIP is a ligand for both VIPR1 and VIPR2, which are expressed in MDA-MB-231 cells. In our transwell migration study, silencing *VIPR2* suppressed the VIP-induced migration to levels similar to that in unstimulated MDA-MB-231 cells. In the scratch wound closure assay, two siRNAs of different sequences targeting *VIPR2* decreased the migration speed by the same degree. Additionally, treatment with KS-133, a selective VIPR2 antagonist, at concentrations above 100 nM almost completely suppressed cell migration induced by VIP (200 nM) in VIPR2-overexpressing MDA-MB-231 cells. Thus, our study demonstrates that VIPR2 modulates cell migration without the need for support from other VIP receptors and implies that VIPR2-dependent downstream signaling may specifically control cell migration induced by VIP.

VIPR2 and VIPR1 are also coupled to a Gαs protein pathway that signals the phosphorylation of ERK (15). In the current study, the onset time of ERK phosphorylation was slightly delayed from 5 min in control cells to 30 min in MCF-7 cells transfected with VIPR2 siRNA, but ERK phosphorylation was less affected by VIPR2 silencing; the peak time of phospho-ERK level was 30 min in both transfected cells and the level was similar at 30 min and 60 min. Thus, VIPR2 may contribute only weakly to ERK activation in these cells or the deficiency of VIPR2 may have been supplemented by pathways through VIPR1 and unknown VIP receptors in MCF-7 cells.

Migration is a polarized cellular process, and the polarity regulates cell mobility in a specific direction (44). Our scratch wound closure assays revealed that the expression of VIPR2 affects VIP-induced protrusive front edge formation, which was regulated by actin remodeling *via* PI3Kγ-WAVE2-ARP2/3 signaling. Moreover, cell migration is regulated by WAVE2-ARP2/3 signaling as well as phosphorylated AKT. AKT phosphorylated through PI3K signaling phosphorylates p21-activated kinase, which promotes myosin II assembly followed by actomyosin contractions for cell migration (45). Indeed, in the scratch wound closure assay, the migration speed, regardless of the polarity and despite the forced straight movement, was also modulated depending on the fluctuation of VIPR2. VIPR2 signaling may confer both the driving force produced by actomyosin contraction and the directional polarity to moving cells to promote cell motility.

This is the first study to examine the newly emerging roles of VIPR2 in cell migration. Our findings conclusively demonstrated that VIPR2 regulates cancer cell migration. Deregulation of VIPR2 signaling may be a potential mechanism underlying the development of cancer cell pathology. We propose that VIP/VIPR2-induced activation of PI3Kγ and consequent production of PI(3,4,5)P_3_ may represent a key step in driving cancer cell motility. Hence, VIPR2 signaling represents a potential therapeutic target for protection against metastatic progression. These findings provide a rationale for the development of VIPR2 antagonists to suppress cancer metastasis *in vivo*.

## Data availability statement

The original contributions presented in the study are included in the article/**Supplementary Material**, further inquiries can be directed to the corresponding authors.

## Author contributions

SA designed the project, performed the experiments, and wrote the manuscript. MY and KO performed parts of the experiments. KS, AH-T, TN, and HH contributed reagents and analysis tools. JAW and YA conceived and coordinated the study and wrote the paper. All authors contributed to the article and approved the submitted version.

## Funding

This work was supported in part by Core Research for Organelle Diseases in Hiroshima University (the MEXT program for promoting the enhancement of research universities, Japan) (S.A.) and collaborative research between Hiroshima University and Ichimaru Pharcos Co. Ltd. (Y.A.). This work was also partially supported by grants from JSPS KAKENHI (grant numbers 20K09905 (S.A.), JP20H00492 (H.H.), 20H03392 (Y.A.), 21K19714 (Y.A.)) and the JSPS Program for Advancing Strategic International Networks to Accelerate the Circulation of Talented Researchers (S2603; H.H.).

## Acknowledgments

We thank Gabrielle White Wolf, PhD, from Edanz (https://jp.edanz.com/ac) for editing a draft of this manuscript.

## Conflict of interest

Kotaro Sakamoto is a full-time employee of Ichimaru Pharcos Co. Ltd. The authors declare that this study received funding from Ichimaru Pharcos Co. Ltd. The funder had the following involvement in the study: interpretation of data.

The remaining authors declare that the research was conducted in the absence of any commercial or financial relationships that could be construed as a potential conflict of interest.

## Publisher’s note

All claims expressed in this article are solely those of the authors and do not necessarily represent those of their affiliated organizations, or those of the publisher, the editors and the reviewers. Any product that may be evaluated in this article, or claim that may be made by its manufacturer, is not guaranteed or endorsed by the publisher.

## Abbreviations

ARP2/3, actin-related protein 2 and 3; cAMP, cyclic adenosine monophosphate; EGFP, enhanced green fluorescent protein; ERK, extracellular signal-regulated kinase; GAPDH, glyceraldehyde-3-phosphate dehydrogenase; GEF, guanine nucleotide exchange factor; GPCR, G protein-coupled receptor; EPAC, exchange protein directly activated by cAMP; PACAP, pituitary adenylate cyclase-activating polypeptide; PI, phosphatidylinositol; PI3K, phosphatidylinositol 3 kinase; PKA, protein kinase A; PTEN, phosphatase and tensin homologue; VIP, vasoactive intestinal peptide; VIPR, vasoactive intestinal peptide receptor; WAVE2, WASP family verprolin homologous protein 2.
