# Supplementary material for: Vasoactive intestinal peptide–VIPR2 signaling regulates tumor cell migration
Source: Front Oncol. 2022 Sep 27;12:852358. doi: 10.3389/fonc.2022.852358 (PMC9550923; doi:10.3389/fonc.2022.852358)
Supplement: Supplementary file 1 [file DataSheet_1.pdf]

Figure S1

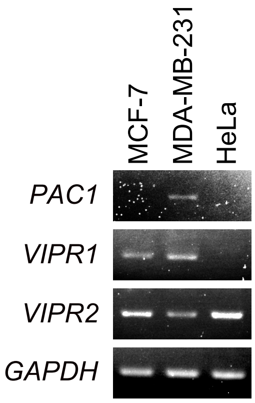


**Figure S1.** *ADCYAP1R1* (*PAC1*), *VIPR1* and *VIPR2* gene expression determined by reverse transcription-PCR analysis in MCF-7, MDA-MB-231 and HeLa cells.

Figure S2

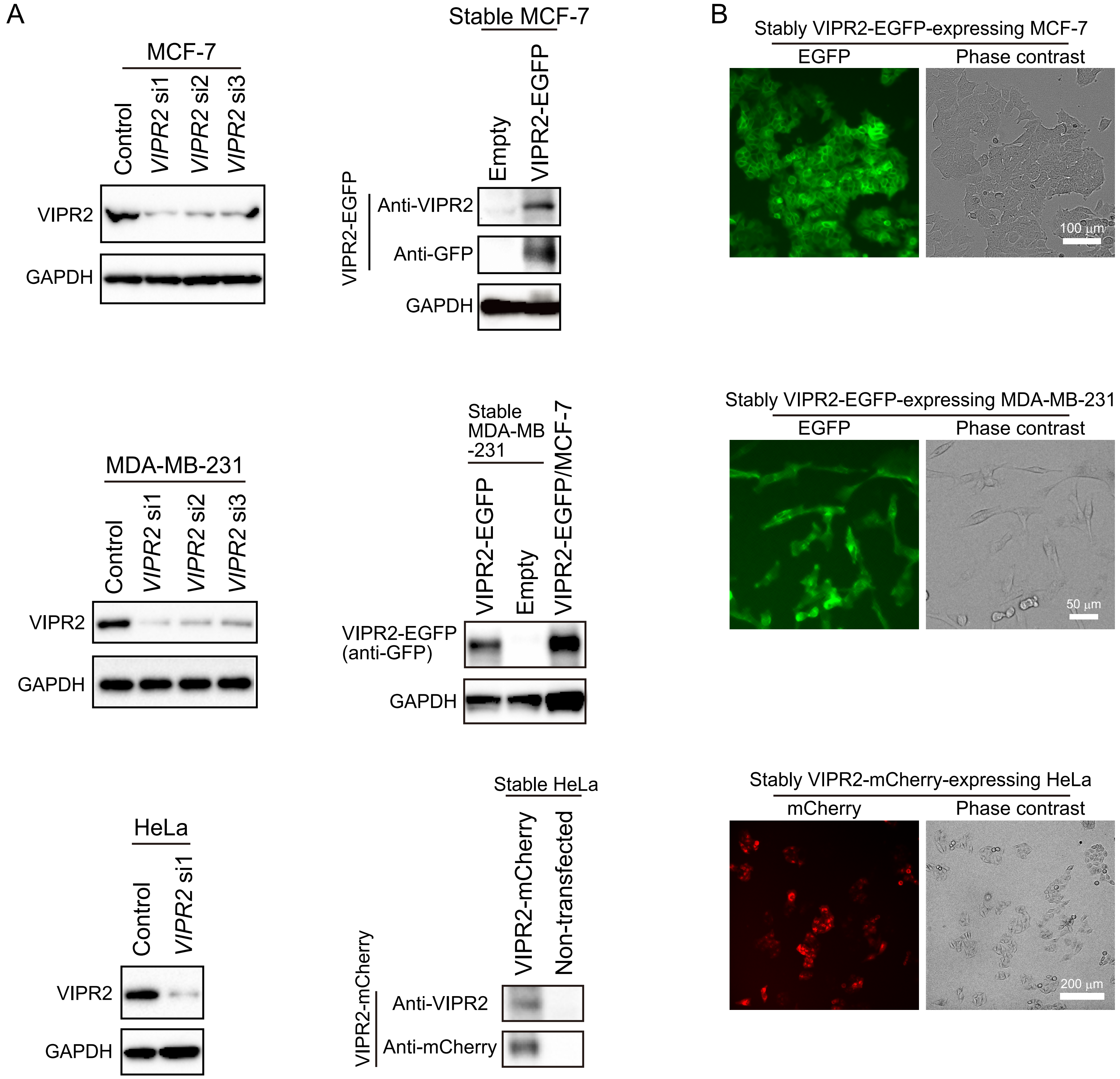


**Figure S2.** The detection of VIPR2 protein in cancer cells (MCF-7, MDA-MB-231 and HeLa cells) transfected with *VIPR2* siRNA or stably expressing exogenous VIPR2 (VIPR2-EGFP or VIPR2-mCherry). A,B. Each Cell line transfected with the indicated siRNA or stably expressing the indicated proteins was analyzed by western blotting using the specific antibodies to the indicated proteins (A) or observed using a fluorescent microscope (B).

Figure S3

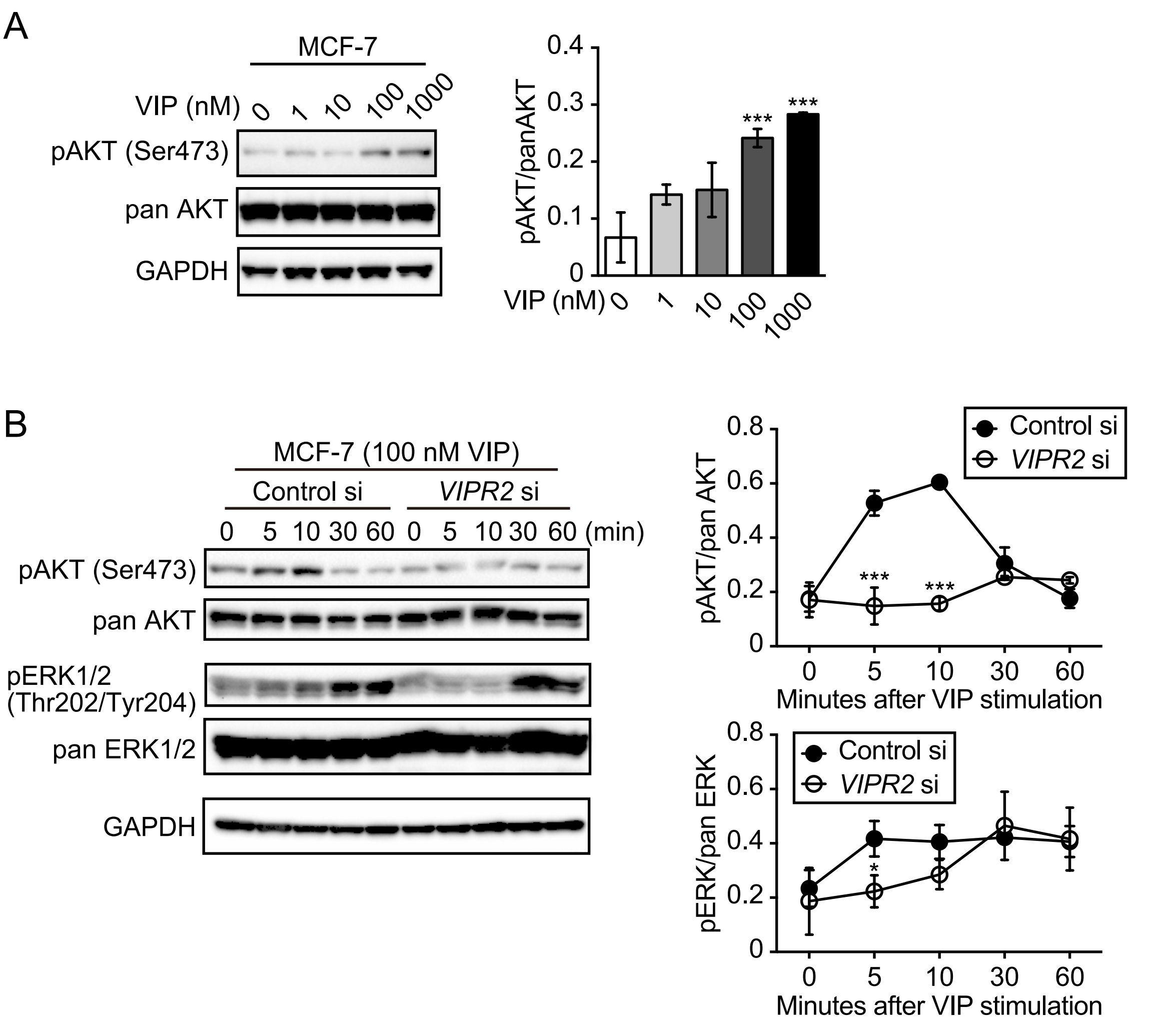


**Figure S3.** VIP-VIPR2 signaling regulates the phosphorylation of AKT in MCF-7 cells. (A) MCF-7 cells were starved for 3 h and then stimulated with the indicated doses of VIP for 10 min. Treated cells were lysed and evaluated by western blotting using specific antibodies for the indicated proteins. A set of representative images is shown. The graph displays the density of pAKT (Ser473) normalized against the corresponding density of the respective pan AKT. The data are presented as means ± SD (n = 3); ***$p < 0.001$ (versus 0 nM VIP; Kruskal–Wallis test followed by Dunn's multiple comparison test). (B) MCF-7 cells transfected with the indicated siRNAs were stimulated with 100 nM VIP for the indicated periods of time. Cell lysates were analyzed by western blotting to detect the indicated proteins. The graphs display the density of pAKT (Ser473) and pERK1/2 (Thr202/Tyr204) normalized against the corresponding density of the respective pan-antibody bands. The data are presented as means ± SD (n = 3); *$p < 0.05$, ***$p < 0.001$ versus respective time-matched controls (Mann-Whitney's U test).

Figure S4

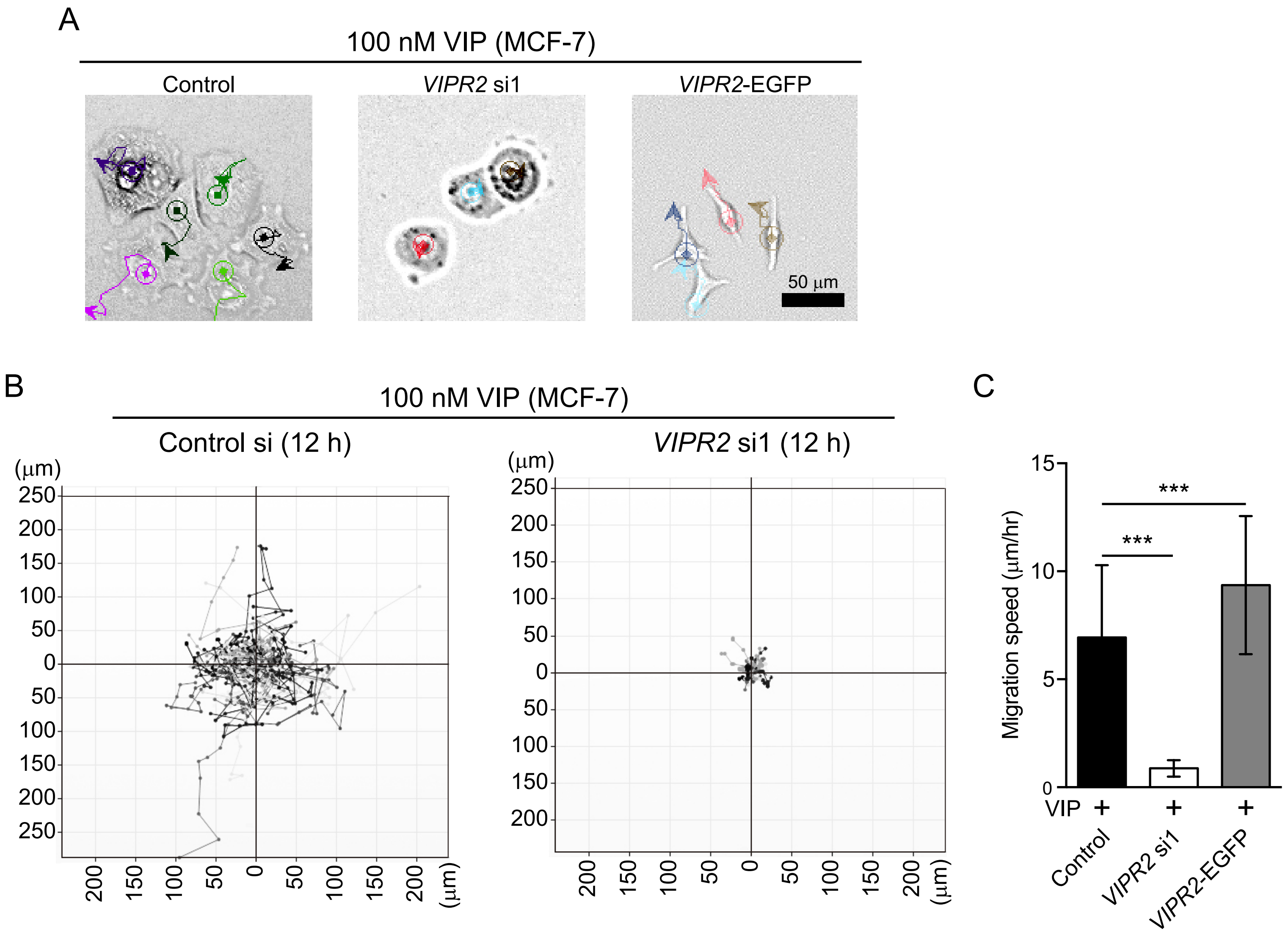


**Figure S4.** Cell motility in *VIPR2*-silenced and VIPR2-expressing MCF-7 cells. (A) MCF-7 cells were transfected with *VIPR2*-EGFP or EGFP plasmids and control siRNA or *VIPR2* siRNA1 and stimulated with 100 nM VIP. Random migration was monitored every 1 h for 12 h after stimulation. Representative track plots are shown. (B) Thirty cells were randomly selected and analyzed. Track plots for 12 h are shown in the graphs. The starting point of each individual cell is located in the center of the diagram. (C) Bar graph shows comparisons of migration speed. Data are presented as means ± SD (n = 60, 31, and 68 in the left-to-right direction on the graph). ***$p < 0.001$ between the indicated groups (Kruskal–Wallis test followed by Dunn's multiple comparison test).
